# Dr. Clarke's Unusual Case of Parturition

**Published:** 1806-07

**Authors:** John Clarke

**Affiliations:** New Burlington Street


### To Dr. BRADLEY.

SIR,

A Circumstance not very common in practice, having lately occurred to me, I beg leave to communicate it through the medium of the Medical and Physical Journal.

I remain, &c.

JOHN CLARKE.

*New Burlington Street, June 6, 1806.*

---

In the month of April last I was desired to attend A. B. in labor of her first child. All the circumstances of the parturition apparently were common, and the placenta followed the fœtus naturally and without any assistance.

Between the birth of the child and the time of the separation of the placenta I applied my hand, as is my invariable custom, to the abdomen, to ascertain if there were a second child, and found the uterus contracted, and with its fundus lying below the level of the navel.

Every thing went on in the usual manner after her delivery; but on the evening of the second day I was suddenly called to her, from her having found something protruding from the os externum.

When I arrived and examined it, I could scarcely distinguish what the substance was, till at length there appeared to be two small, thin, hard bodies, about the size of a wing-quill of a pigeon, and by a little more attention I discovered that they were the legs of a small embryo. I slowly and gently pulled by them, and brought away the whole of a fœtus about the third month of gestation, measuring about three inches from the top of the compressed skull to the extremity of the feet. The whole was extremely putrid and abominably offensive to the smell. The brain must have been discharged, as the rudiments of the bones of the two sides of the head were in close contact with each other; the skin and muscles were loose, flabby, and almost reduced to the state of pulp. The whole of the fœtus weighed, as I conjecture, about three drachms, or half an ounce. It was too putrid to admit of being preserved, I therefore burned it, as I generally do substances connected with pregnancy which are discharged from the uterus, if they are not likely to be useful for

 instruction instruction as a preparation, and are not of a nature sufficiently important to be deposited in a church-yard*

Cases of this nature have often given occasion to the opinion of the possibility of superfœtation from two fœtuses of very different sizes having been expelled from the uterus at the same time. In such cases however the smaller is never found to be living. Hence it amounts almost to demonstration that the two were formed at one impregnation; but from some disease of one of them, or from some derangement of its placenta, it had died at an early period of pregnancy and had remained in the uterus during the period of utero-gestation till the other living child was expelled.

This was the fact here, and the only peculiarity in the case consists in the blighted embryo between the third and fourth month of pregnancy, coming away two days after the living child.

As it did not come away with the placenta, I presume that it lay in the vagina till it began to project through the os externum, having been most probably expelled from the uterus with the placenta. If it had been larger, it would have been forced away from the vagina also; but from its minute bulk it escaped by the edge of it. It is likely that the remains of the placenta of the blighted fœtus made part of the general placenta, as is most frequently the case in twins, since nothing of that nature came away afterwards.

I have in my collection a blighted fœtus, where the circumstances of the two children were the same; but this had advanced as far as the fifth month, and not being very putrid, admitted of being preserved in spirits. The head was in like manner flattened, and the brain squeezed. The abdomen and chest had also been compressed by the growth of the living child.

* The origin of burning the placenta and that part of the funis umbilicalis which separates from the navel of a child, was probably a Pagan sacrifice to Juno Lucina, as it is of great antiquity. This is farther confirmed by a superstitious notion very prevalent among the lower ranks, that it is unlucky for a child that the portion of funis which separates from the navel should touch the earth. At any rate the practice of burning such substances, instead of putting them into the ground, or throwing them into a privy, is proper, because if they should be found in such situations undissolved, some suspicions of a concealed pregnancy might fall on an innocent woman.

